# 3DCryoHolder: a new open access 3D printable system to store and transport silicon nitride membranes under cryogenic conditions for spectromicroscopy at low temperature

**DOI:** 10.1107/S1600577524010919

**Published:** 2025-01-01

**Authors:** Antonio Dominguez-Alfaro, Carlos Sanchez-Cano

**Affiliations:** a Polimero eta Material Aurreratuak: Fisika, Kimika eta Teknologia, Kimika Fakultatea, Euskal Herriko Unibertsitatea UPV/EHU, Donostia-San Sebastian20018, Spain; b Donostia International Physics Center Paseo Manuel de Lardizabal 4 Donostia-San Sebastian20018 Spain; c Ikerbasque Basque Foundation for Science Plaza de Euskadi 5 Bilbao48009 Spain; SESAME, Jordan

**Keywords:** X-ray cryo-spectromicroscopy, cryopreserved samples, storage and transport, 3D printing

## Abstract

Data acquisition under cryogenic conditions allows one to avoid unwanted damage caused by beam irradiation. This is particularly important for the study of biological samples at hard X-ray, micro- or nano-probe beamlines, which focus synchrotron radiation to small beam sizes with extremely high flux densities. Sample preparation methods for cryopreserved specimens have been adapted from electron microscopy, and include the use of silicon nitride membranes as they are easy to handle and possess low X-ray absorption. Yet, currently there are no commercially available methods for the storage and transport of silicon nitride membranes under cryogenic conditions. Here, we introduce and provide the design files of 3DCryoHolder, a system that can be 3D printed in-house for the correct storage and transport of multiple silicon nitride membranes under cryogenic conditions, and is compatible with all commercial plunge-freezing instruments.

## Abstract 2

A system (3DCryoHolder) has been developed to store and transport silicon nitride membranes under cryogenic conditions for X-ray spectromicroscopy measurements at synchrotron research facilities. The system is 3D printable and compatible with different plunge-freezing makers, and is provided here for free use by the community.

## 1. Introduction

X-ray measurements requiring long acquisition times can lead to unwanted alterations on the chemical and structural properties of the samples studied. This is normally caused by ionization or heat-related processes, and is a large problem when probing delicate samples from biological origins such as organs, tissues or cells (Schneider *et al.*, 1995 ▸; Garman & Weik, 2021 ▸; Lawrence Bright *et al.*, 2021 ▸). Moreover, while the rise of current state-of-the-art beamlines have permitted one to examine biological systems with unprecedented resolutions at the micro- or nanometre scales, the extreme focusing needed for such analysis has led to beams with high flux densities that increase the damage caused to the sample during the acquisition. Such beam-related alterations can reduce the significance of the results obtained (Bras *et al.*, 2021 ▸) and require methods to control it (Townsend *et al.*, 2022 ▸; Quinn *et al.*, 2024 ▸).

Experimental acquisition under cryogenic environments helps to reduce unwanted damage caused by intense irradiation of biological samples because low temperatures compensate extreme heat increases and vitrification of water decreases the movement of radicals in non-dehydrated samples (Li *et al.*, 2024 ▸). As such, cryogenic conditions are largely used to study biological samples with electron microscopy (EM) techniques. Multiple synchrotron beamlines have also introduced (or are planning) the ability to work under cryogenic conditions into their capabilities. Nevertheless, preparation of cryopreserved samples is intricate and requires the use of specific protocols to avoid unwanted damage due to the formation of water crystals. Such protocols are well established for EM techniques and can be modified with ease for the preparation of samples for analysis at synchrotron radiation facilities. For example, cells can be grown on top of EM grids or silicon membranes before cryofixation and cryopreserved by rapid immersion on liquified ethane (or similar) (Bissardon *et al.*, 2019 ▸; Finney & Jin, 2015 ▸; Jin *et al.*, 2017 ▸). In most laboratories, this is carried out using instruments like the Leica EM-GP, the Thermofisher Scientific Vitrobot or the Gatan Cryoplunge. Although, there are more modern and automated alternatives directly designed for cryo-EM or correlative light-electron microscopy (CLEM) approaches (Koning *et al.*, 2022 ▸; see also https://www.linkam.co.uk/cryogenium and https://www.sptlabtech.com/products/chameleon/chameleon).

Still, cryopreserved samples need to be stored and transported under the right conditions, ideally using systems that are compatible with the plunger instrument used. For EM grids, holders are commercially available and compatible with the Leica, Thermofisher and Gatan instruments, and practical storage and transport devices have been designed by the community for 3D printing and are free to use by anybody (Hamaguchi & Yonekura, 2020 ▸). However, there are no commercially available holders for the storage and transport of silicon nitride membranes. Moreover, although a holder has been designed for the storage of the membranes, it is only compatible with Leica and Gatan instruments (Bissardon *et al.*, 2019 ▸), and to the best of our knowledge there are no devices designed for their transport.

Here, we introduce 3DCryoHolder, a new method for the correct storage and transport of multiple silicon nitride membranes under cryogenic conditions (up to 16 per each complete system), which is compatible with all three Leica, Thermofisher and Gatan instruments. Design files are also provided, so the devices can be produced easily in-house using a fused deposition modelling (FDM) 3D printer.

## 2. Design of the storage and transport system

**Figure 1:**
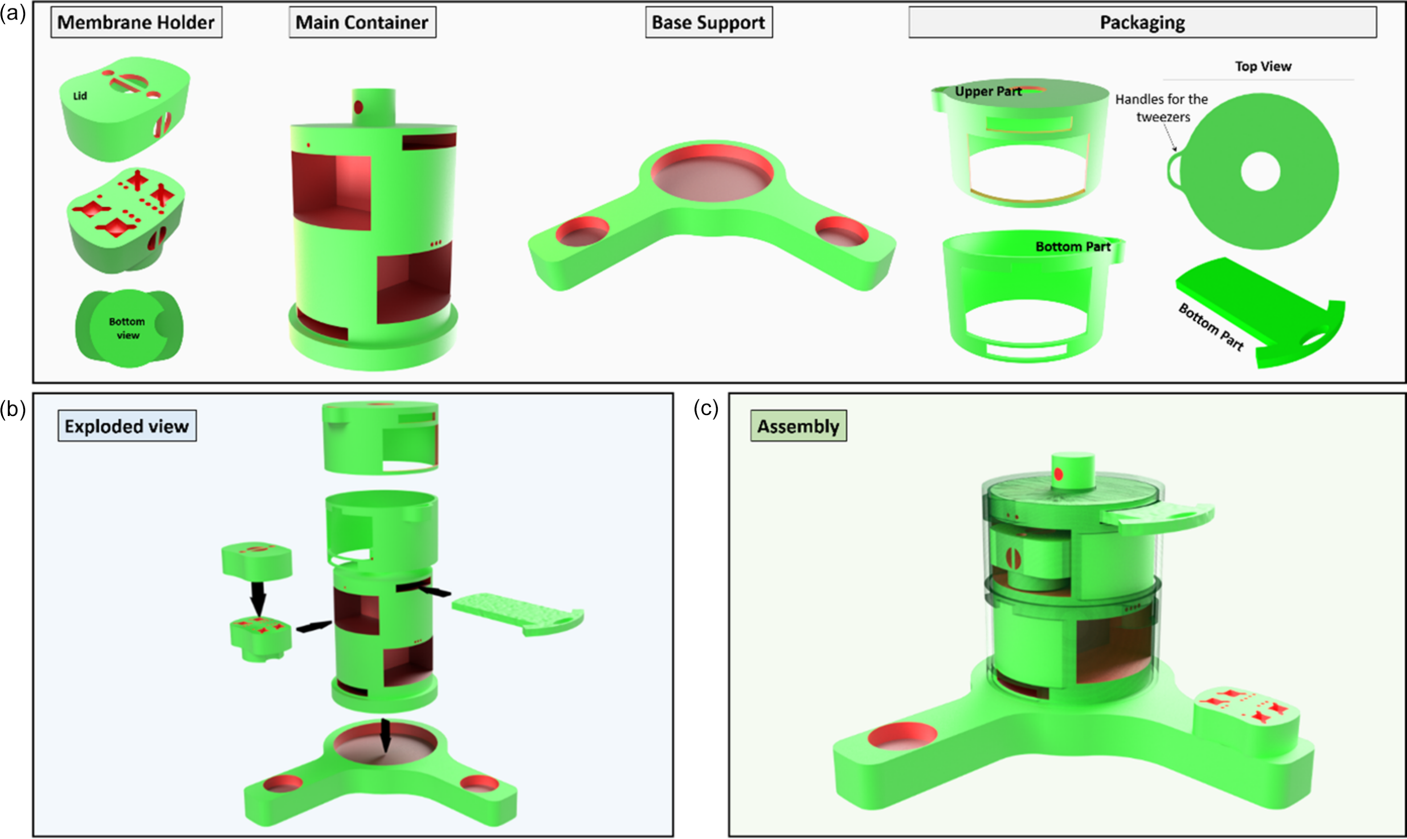
(*a*) A schematic illustration of the different components of 3DCryoHolder (membrane holder, main container and base support) for the storage and transport of silicon nitride membranes under cryogenic conditions, (*b*) an exploded view, and (*c*) an assembly of all the components.

3DCryoHolder was designed using the *Autodesk Inventor* CAD/CAM software package (https://www.autodesk.com/eu) with four main specifications in mind: (1) capacity to hold and transport securely multiple carbon-framed silicon nitride windows, (2) allow transport and identification of different samples within the same system, (3) compatibility with all three main plunge-freezer commercial devices (Leica EM-GP, Thermofisher Scientific Vitrobot and Gatan Cryoplunge), and (4) easy manipulation under cryogenic conditions including loading and unloading of the samples within the cryoholder. Following these specifications, we generated a multi-component system with three main parts (Fig. 1 ▸): (i) a membrane holder that can be placed in plunge-freezing devices to permit safe transfer of the samples; (ii) a main container for the storage and transport of multiple membrane holders under cryogenic conditions; and (iii) a base support to facilitate the loading and unloading of the container, and the manipulation of membrane holders under cryogenic vapour phase.

The membrane holder is a solid block containing four slots for the storage of 5 mm × 5 mm squared carbon-framed silicon nitride membranes, but can be easily modified to accommodate membranes of different sizes and shapes. Each of the slots is numbered using dots (1 •, 2 ••, 3 ••• and 4 ••••) to allow sample identification, and enlarged near the surface to facilitate access to tweezers during loading and unloading of the membranes (while restricting membrane movement once loaded). The holder also has a slit connector at the bottom to permit correct positioning at the plunge freezer and is bean-shaped to allow its use on the Thermofisher Scientific Vitrobot. The confinement of the membranes during transport is secured using a lid, which contains specific areas to allow putting on/taking off the lid or handling whole holders using tweezers.

Following the same approach, it might be possible to modify cryo-EM boxes to accommodate silicon nitride windows. However, it is likely that such systems would only be able to store two or three silicon nitride membranes maximum, instead of the four that can be stored in each of the membrane holders described here.

The main container is designed to keep the membrane holders safe during storage and transport under cryogenic conditions. It is composed of four compartments capable of storing a membrane holder each and positioned on a 2 × 2 array (top and bottom) in an alternated configuration. Each of the compartments is numbered using dots (in the same manner as the membrane slots within the holder) for the identification of the holder stored. Two individual sleeves placed over the container allow one to control access to the individual compartment desired, and specific holes in the sleeves permit locking them in place using inserts (which go through cavities within the body of the container). Moreover, both the sleeves and inserts have large grips to facilitate their handling with tweezers, while the top of the container can be tied to assist recovery from cryogenic storage vessels or dry shippers.

Finally, the base support holds and secures the main container, allowing easy recovery and manipulation of the membrane holders and membranes under liquid nitro­gen vapour phase.

## 3. Production and use of the storage and transport system

The 3DCryoHolder system was fabricated in polylactic acid (PLA) thermoplastic using a commercial FDM 3D printing device. This combination of CAD/CAM with 3D printing provides rapid prototyping, secures low cost and opens new opportunities for the production of custom-made devices tailored for the exact needs of specific research communities (Tiersch & Monroe, 2016 ▸).

Our selection of 3D printing technology and production material was directed by considerations based on previous reports showing the production from simple and inexpensive devices to more complex ones (Tiersch & Monroe, 2016 ▸; Childress et al., 2023 ▸). Moreover, some examples of systems for the storage and transport of cryo-samples in EM grids for EM-related techniques are already present in the literature and open to use (Hamaguchi & Yonekura, 2020 ▸).

In particular, 3D printing using FDM approaches is ideal for cryogenic applications and preferred over stereolithography (Tiersch & Monroe, 2016 ▸). FDM-printed objects are porous, with low density, low thermal mass and a high strength-to-weight ratio. Equally, thermoplastic polymers are better for cryogenic applications than thermoset polymers or light-triggered polymers (which show high strength and thermal stability but are brittle under cryogenic conditions) (Tiersch & Monroe, 2016 ▸; Chen *et al.*, 2021 ▸). In principle, polyether ether ketone would be the ideal material for the production of the 3DCryoHolder system. However, the high melting temperature of this polymer makes its use difficult in certain cases as it requires access to FDM printers more advanced and expensive than those used with other thermoplastics. PLA and acrylo­nitrile butadiene styrene (ABS) are the other thermoplastics most commonly used, and are accepted as good alternative materials to print parts for cryogenic environments (Tiersch & Monroe, 2016 ▸; Bartolomé *et al.*, 2017 ▸; Weiss *et al.*, 2015 ▸). In our case, PLA was chosen for our system as it is considered superior to ABS. PLA has a lower glass transition (*T*_g_) temperature, so it can be extruded at lower temperatures and cools down in a more uniform fashion. This prevents the build-up of internal stresses in the object, which could cause distortions when exposed to cryogenic conditions (Selva Priya *et al.*, 2019 ▸; Vaught *et al.*, 2023 ▸). On the other hand, ABS can suffer large contractions and failure at cryogenic temperatures due to thermic stress (although, this can be reduced by careful cooling of the ABS part through temperature gradients) (Weiss *et al.*, 2015 ▸).

The different parts of 3DCryoHolder were produced on an Ultimaker 2+ Connect printer, using the parameters described in Table 1 ▸. Skirts were used around all of the different parts during the printing process to help plate adhesion, but supports were only used to produce the main container and the sleeves around it. Parts were not cured or polished after printing, and could be used straight after removal of the raft and supports from the main structure. Files for the printing of each of the components of the system can be found in the supporting information (see design files S1–S7).

**Figure 2:**
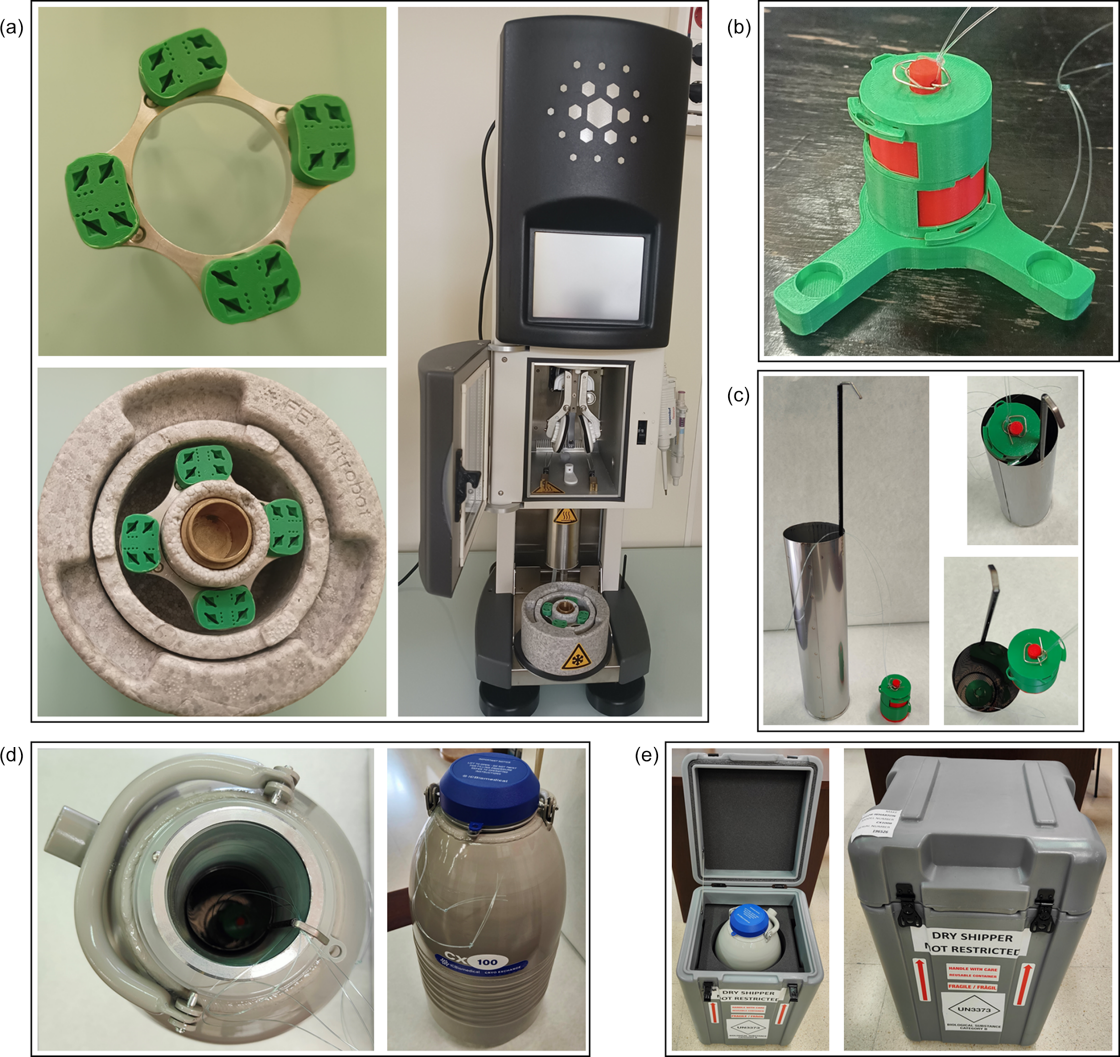
(*a*) Fitting of 3DCryoHolder holders at a Thermofisher Scientific Vitrobot, (*b*) a fully assembled 3DCryoHolder system, (*c*) fitting of 3DCryoHolder systems at the inner cannister of a standard commercial dry shipper, (*d*) positioning of the inner cannister inside the dry-shipper dewar and (*e*) placement of the dry shipper in a commercial rigid case for transport.

The 3DCryoHolder storage and transport system generated has already been tested in a number of experiments at hard X-ray cryo-nanoprobes (Skiba *et al.*, 2024 ▸; Santiago-Arcos *et al.*, 2024 ▸; Terenzi & Cano, 2024 ▸). In our experience, the system is simple to use, as it contains multiple areas to facilitate the grip and access of tweezers. This makes handling of all the operations required possible with two tweezers (Video S1 of the supporting information). 3DCryoHolder is prepared to be stored in liquid nitro­gen and transported safely under vapour-phase cryogenic conditions at the inner cannister of dry shippers. The different parts of the system withstand cryogenic cooling well, both immersion in liquid nitro­gen and staying under liquid nitro­gen vapour phase. Moreover, the membrane holder is compatible with Thermofisher Scientific Vitrobot plunge freezers, and four of them can be easily placed near the freezing area of the instrument to secure a rapid and safe transfer of the sample (Fig. 2 ▸). Finally, the container cylinders facilitate safe storage on cryogenic vessels without further modification than the use of a string to assist recovery from cryogenic vessels, and also the transport and delivery of the samples using standard dry shippers.

## 4. Conclusions

We report here the design and production of 3DCryoHolder using conventional FDM 3D printing hardware, a multi-component system for the storage and transport of samples immobilized in silicon membrane substrates under cryogenic conditions. The system fulfils a current necessity within the field of X-ray spectromicroscopy, is compatible with all three main plunge-freezing devices (Leica EM-GP, Thermofisher Scientific Vitrobot and Gatan Cryoplunge) and has been demonstrated to work as intended during a number of experiments (Skiba *et al.*, 2024 ▸; Santiago-Arcos *et al.*, 2024 ▸; Terenzi & Cano, 2024 ▸). As such, we provide the design files within the supporting information of this article to facilitate in-house fabrication at any laboratory interested.

## Supplementary Material

Design files S1-S7. DOI: 10.1107/S1600577524010919/kam5003sup1.bin

Video S1. DOI: 10.1107/S1600577524010919/kam5003sup2.mp4

## Figures and Tables

**Table 1:** 3D printing parameters used during production of 3DCryoHolder

Layer height	0.15 mm
Line width	0.35 mm
Wall thickness	0.7 mm
Infill density	100%
Printing temperature	210°C
Building-plate temperature	70°C
Printing speed	55 mm s^−1^
